# Improving Characterization of Understudied Human Microbiomes Using Targeted Phylogenetics

**DOI:** 10.1128/mSystems.00096-20

**Published:** 2020-02-25

**Authors:** Bruce A. Rosa, Kathie Mihindukulasuriya, Kymberlie Hallsworth-Pepin, Aye Wollam, John Martin, Caroline Snowden, William Michael Dunne, George M. Weinstock, Carey-Ann D. Burnham, Makedonka Mitreva

**Affiliations:** a McDonnell Genome Institute at Washington University, St. Louis, Missouri, USA; b Department of Medicine, Washington University School of Medicine, St. Louis, Missouri, USA; c Department of Pathology & Immunology, Washington University School of Medicine, St. Louis, Missouri, USA; d The Jackson Laboratory for Genomic Medicine, Farmington, Connecticut, USA; Qingdao Institute of Bioenergy and Bioprocess Technology, Chinese Academy of Sciences

**Keywords:** HMP, genome, human microbiome, microbiome, resource

## Abstract

Whole-genome bacterial sequences are required to better understand microbial functions, niche-specific bacterial metabolism, and disease states. Although genomic sequences are available for many of the human-associated bacteria from commonly tested body habitats (e.g., feces), as few as 13% of bacterium-derived reads from other sites such as the skin map to known bacterial genomes. To facilitate a better characterization of metagenomic shotgun reads from underrepresented body sites, we collected over 10,000 bacterial isolates originating from 14 human body habitats, identified novel taxonomic groups based on full-length 16S rRNA gene sequences, clustered the sequences to ensure that no individual taxonomic group was overselected for sequencing, prioritized bacteria from underrepresented body sites (such as skin and respiratory and urinary tracts), and sequenced and assembled genomes for 665 new bacterial strains. Here, we show that addition of these genomes improved read mapping rates of Human Microbiome Project (HMP) metagenomic samples by nearly 30% for the previously underrepresented phylum *Fusobacteria*, and 27.5% of the novel genomes generated here had high representation in at least one of the tested HMP samples, compared to 12.5% of the sequences in the public databases, indicating an enrichment of useful novel genomic sequences resulting from the prioritization procedure. As our understanding of the human microbiome continues to improve and to enter the realm of therapy developments, targeted approaches such as this to improve genomic databases will increase in importance from both an academic and a clinical perspective.

**IMPORTANCE** The human microbiome plays a critically important role in health and disease, but current understanding of the mechanisms underlying the interactions between the varying microbiome and the different host environments is lacking. Having access to a database of fully sequenced bacterial genomes provides invaluable insights into microbial functions, but currently sequenced genomes for the human microbiome have largely come from a limited number of body sites (primarily feces), while other sites such as the skin, respiratory tract, and urinary tract are underrepresented, resulting in as little as 13% of bacterium-derived reads mapping to known bacterial genomes. Here, we sequenced and assembled 665 new bacterial genomes, prioritized from a larger database to select underrepresented body sites and bacterial taxa in the existing databases. As a result, we substantially improve mapping rates for samples from the Human Microbiome Project and provide an important contribution to human bacterial genomic databases for future studies.

## Abstract (precis)

The human microbiome plays a critically important role in health and disease, but current understanding of the mechanisms underlying the interactions between the varying microbiome and the different host environments is lacking. Having access to a database of fully sequenced bacterial genomes provides invaluable insights into microbial functions, but currently sequenced genomes for the human microbiome have largely come from a limited number of body sites (primarily feces), while other sites such as the skin, respiratory tract, and urinary tract are underrepresented, resulting in as little as 13% of bacterium-derived reads mapping to known bacterial genomes. Here, we sequenced and assembled 665 new bacterial genomes, prioritized from a larger database to select underrepresented body sites and bacterial taxa in the existing databases. As a result, we substantially improve mapping rates for samples from the Human Microbiome Project and provide an important contribution to human bacterial genomic databases for future studies.

## INTRODUCTION

As sequencing technology improves, the increased practicality of genomic analysis has allowed for new inquiries into the workings of the human microbiome. Already, research has characterized the microbial communities of diverse body sites and elucidated the role that microbiota may play in conditions including type 2 diabetes, cardiovascular disease, obesity, cancer, and autism (1–4). Future research aspires to manipulate microbial communities as a prophylactic or therapeutic approach to prevent and/or control various infectious and disease states.

The data for these microbiome studies generally take the form of either 16S rRNA gene sequences or metagenomic shotgun sequencing. The former is frequently used for its efficiency and affordability, requiring only marker genes to be known in order to align and annotate reads. However, exclusive use of this type of data has become limiting to researchers who wish to understand a more complete picture of microbial functions and metabolism within a niche. Metagenomic shotgun sequencing can provide answers to these kinds of question, but it relies upon the use of a reference genome for either alignment or k-mer comparison of genomes intermixed from hundreds to thousands of different taxa (5).

In the past, there have been several efforts to compile a database of microbial genomes. Most notably, the MetaHIT (6) and Human Microbiome Project (HMP) (7) gene catalogs made available catalogs based upon over a hundred European and American samples, respectively. In 2014, Li et al. amassed these data in addition to data from other international studies to create an integrated gene catalog for the human gut microbiome (IGC) (8). This catalog contained almost 10 million nonredundant genes, but it still remains far from comprehensive. Most of these species were cultured from healthy patients whose microbial communities likely differ from those of disease states (9). Additionally, a disproportionate amount of the available reference genomes belong to gut and urogenital microbiota, neglecting other taxa that might be pertinent to studies of skin, vaginal, or respiratory microbiomes (10). In fact, a 2014 study examining the effect of genomic database composition on metagenomic read mapping found that with existing databases, read mapping varied significantly between body sites. The highest-performing areas (posterior fornix) mapped as many as 92% of reads, but samples in the lowest-performing areas (skin) mapped as low as 13% (11). Finally, microbiome composition varies not only between sites on an individual but also between individuals in diverse environments, suggesting that a broader spectrum of reference genomes may be necessary for broad applicability of microbiome analytic techniques (12).

As studies continue to unearth the variety of microbiomes found in multiple countries or a single individual, as well as the importance of tracing new bacterial strains in the setting of an outbreak, it becomes increasingly important to populate the bacterial phylogeny while representing diverse body habitats by generating high-quality reference genomes to publicly available databases. Several recent studies have contributed to human microbiome genomic resources, including two large studies of the human gut microbiome (13, 14), and a study that assembled genomes across a variety of samples, from metagenomic shotgun sequences (15). In our study, the sequencing and analyses of 10,000 full-length 16S rRNA gene sequences identified underrepresented phylogenetic lineages and sequences from underrepresented body sites, 665 of which were prioritized, sequenced, and assembled at a whole-genome level. Unlike a recent metagenomic study with similar goals (15), the samples here represent isolated bacterial cultures representing purified single strains of bacteria, providing higher confidence in the genomic assemblies. Analyses of the 665 genomes illuminated the importance of taxonomic prioritization in new genome discovery and demonstrated the effects of an unbiased database on microbiome characterization. The approach and the resources are of importance at both the academic and clinical research levels.

## RESULTS AND DISCUSSION

### Composition of sequenced genomes.

**FIG 1:**
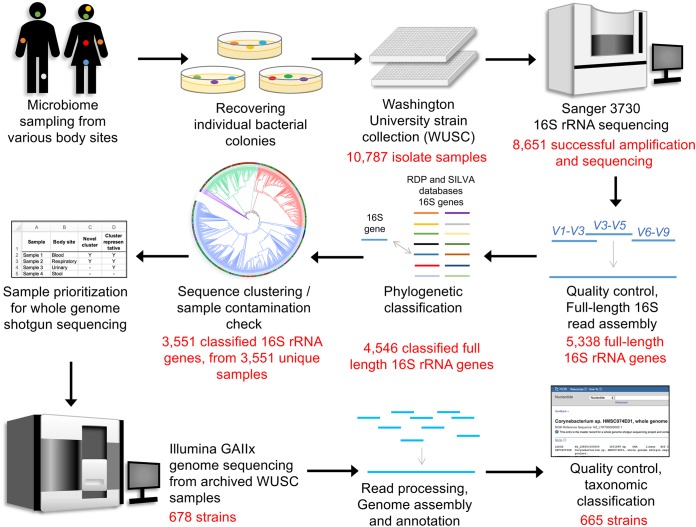
Flow chart diagram of overall sample production and prioritization procedure.

The complete Washington University Strain Collection (WUSC), comprising 10,787 bacterial taxa isolated from clinical samples, underwent 16S rRNA gene sequencing. A total of 4,546 full-length 16S rRNA gene sequences were phylogenetically classified following sequencing, assembly, quality control, and annotation (Fig. 1). After controlling for contaminated samples and selecting single bacterial representatives per original sample, 3,551 full-length 16S rRNA gene sequences (see Table S1 in the supplemental material) were interrogated based on (i) their 16S classification, prioritizing novel or underrepresented taxonomic groups in existing public databases; (ii) their body site source, prioritizing underrepresented body sites (such as blood, peritoneal fluid, and wounds); and (iii) the quality and availability of the source DNA. The analysis resulted in 665 strains to be selected for whole-genome sequencing, assembly, and annotation, representubg 15% of the candidate samples initially screened for novelty. More than 89% (594/665) of assembled genomes were over 99% complete (according to BUSCO [16]), and over 98% of samples (655) had genome coverage depths of over 50×. Table S2 contains, for each of the 665 genomes, accession information; complete phylogenetic classification; body site source data; and completeness, assembly, and annotation statistics.

10.1128/mSystems.00096-20.1 TABLE S1 Full taxonomy, isolate source, and sequence cluster information for all 3,551 unique 16S samples. Download Table S1, XLSX file, 0.4 MB. Copyright © 2020 Rosa et al. 2020 Rosa et al. This content is distributed under the terms of the Creative Commons Attribution 4.0 International license.

10.1128/mSystems.00096-20.2 TABLE S2 NCBI accession information and hyperlinks, full taxonomy, isolate source data, sequence cluster identifications, and details of assembly and annotation statistics for all 665 sequenced genome samples. Download Table S2, XLSX file, 0.2 MB. Copyright © 2020 Rosa et al. 2020 Rosa et al. This content is distributed under the terms of the Creative Commons Attribution 4.0 International license.

**FIG 2:**
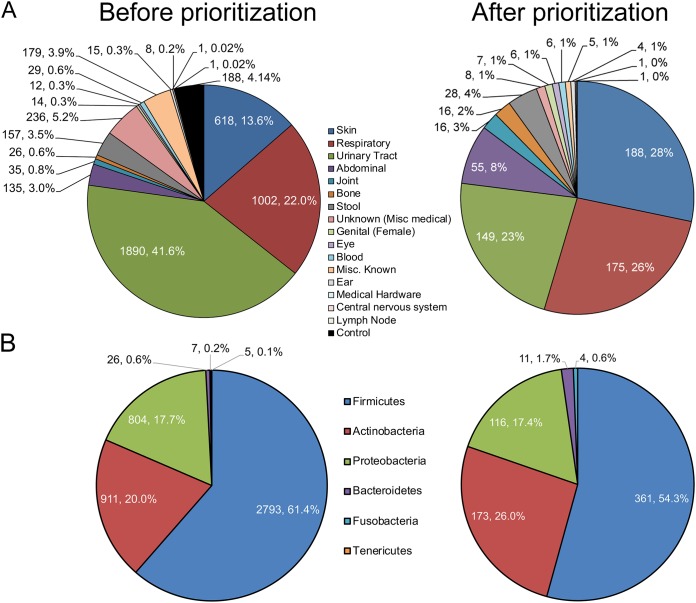
Composition of isolates before (left) and after (right) prioritization. (A) Isolate categorization by body habitat. (B) Isolate categorization by phylogeny at a phylum level.

Before and after prioritization, samples were taken from over 15 different body sites, with the largest taxonomic representation drawn from skin, respiratory tract, and urinary tract cultures (Fig. 2A). Prioritization caused a relative increase of at least 300% in sparsely sampled areas such as joint and bone, while relative representation of samples from medical hardware, urinary tract, and unknown locations was reduced, perhaps due to the homogeneity of bacterial communities that has previously been observed (17, 18). The fecal microbiota was underrepresented among preprioritized and prioritized samples, since it is the best characterized of the body locations (10). Note that “unknown” samples were from a specific sample type (such as wound or abscess), but these were not assigned a specific body site in the metadata. Additionally, the prioritization considered phylogenetic clustering, with the intention to avoid overselecting the same strains of bacteria, regardless of species or sample site origin (99% identity over 95% length). This resulted in a wider array of unique taxa being sequenced than would have been selected without considering phylogeny.

The majority of samples before and after prioritization were part of the *Firmicutes* phylum, followed in abundance by the *Actinobacteria* and *Proteobacteria* phyla. *Bacteroidetes*, *Fusobacteria*, and *Tenericutes* were sparsely represented in samples (Fig. 2B). After prioritization, relatively fewer *Firmicutes* samples were chosen for sequencing, since studies historically have identified primarily taxa in the *Bacteroidetes* and *Firmicutes* phyla, which tend to be more abundant in the gut (10). Instead, the relative representation of *Actinobacteria* in samples to be sequenced increased after prioritization. *Actinobacteria* are an important component of several different body location microbiomes, including skin folds (19) and the poorly characterized eye mucosal microbiome (20). In addition to *Fusobacteria*, it also comprises a notable part of oral microbiomes such as that of the tongue dorsum or supragingival plaque (20). As the literature regarding these areas continues to expand, further characterization of their microbiomes will require genomic libraries including more taxa within the *Actinobacteria* or *Fusobacteria* phylum, which may not have appeared in earlier gut microbiome studies. This is becoming increasingly important as these taxa have been implicated in a number of health and disease states, such as colon cancer (21–23).

**FIG 3:**
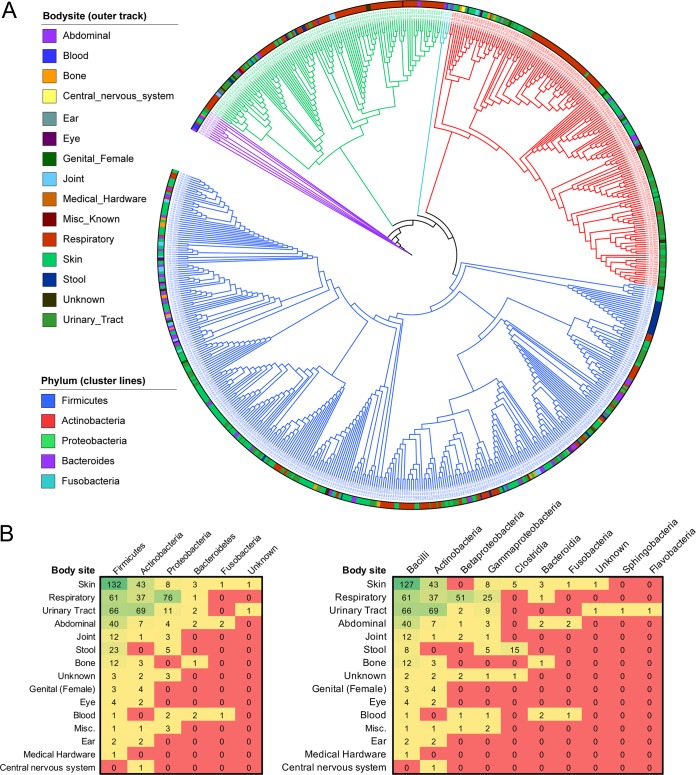
Grouping of the sequenced WUSC bacterial genomes based on body site, phylum, and class. (A) WUSC strains clustered based off 16S read sequence similarity. Phylum is indicated by dendrogram branch color, while body habitat is indicated by the colored bars around the periphery of the image. (B) WUSC strains classified by body site and phylogeny at a phylum (left) or class (right) level. Counts indicate the number of strains in each category; color indicates the level of representation in each category, with green representing high counts and red representing low or none.

Clustering of the prioritized isolates by 16S rRNA gene read counts showed clear assortment by phyla (Fig. 3A). Some body sites tended to cluster within phyla; most notably, respiratory tract samples were primarily within the *Proteobacteria* phylum, while skin samples clustered within the *Firmicutes* phylum and *Bacilli* class (Fig. 3B). It is interesting that these phyla are not those predominantly associated with the respiratory tract or skin, respectively. Studies suggest that the principal taxa in the respiratory tract lie within the *Bacteroidetes* and *Firmicutes* phyla (24), while skin samples vary greatly, but frequently contain *Actinobacteria*, *Proteobacteria*, or bacteria within the *Staphylococcaceae* family of *Firmicutes* (19). The differences between our data clusters and the normal skin or respiratory tract communities reflect the efforts of our prioritization method to obtain sequences distinct from the known biological landscape. These sequences may become particularly important in efforts to characterize disease-state deviations from the normal microbiome: for example, research suggests that asthma may be associated with enrichment of bacteria in the *Proteobacteria* phylum, within which our prioritized respiratory samples tended to cluster (25). Characterization of such deviate taxa, as opposed to those that fall within the norm, may give us better resolution in our view of how physiological processes associate with changes in diverse microbiota.

### Effect of database sequence augmentation on characterization of metagenomics shotgun sequences.

**FIG 4:**
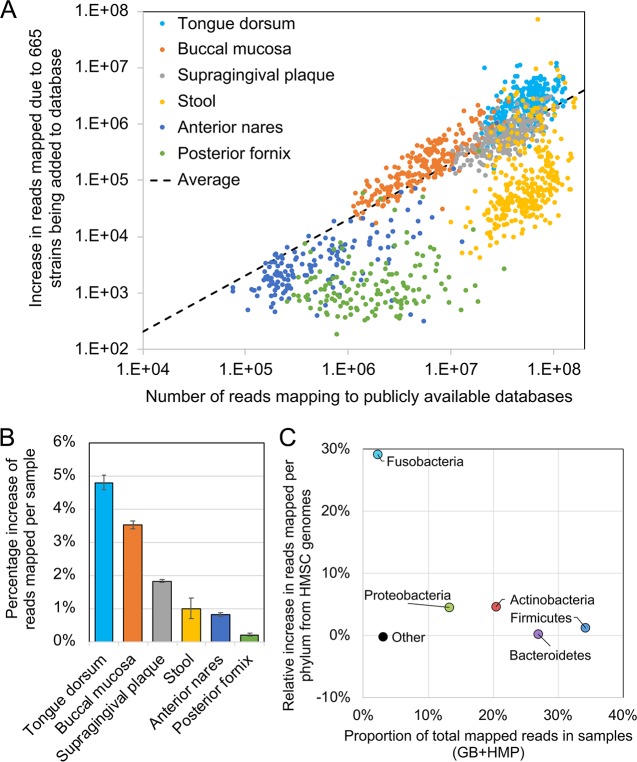
Increased characterization of metagenomic shotgun sequences by addition of genomes of novel phylogenetically distinct strains. (A) Relative improvement of read mapping rates from HMP samples to the improved genome database. (B) Number of total mapped reads per sample after novel reference genome sequence inclusion versus the absolute increase in mapped reads after versus before novel sequence inclusion. Samples are colored by body site. The dotted line represents the average relationship between total number of mapped reads and absolute increase in mapped reads after novel reference genome sequence inclusion. (C) The proportion of total mapped reads after novel sequence inclusion attributable to a given phylum versus the relative increase in reads mapped for that phylum after novel sequence inclusion. Phyla are represented as differently colored dots, with names given on the chart.

Compared to publicly available genome databases alone (HMP plus GenBank; 4,383 strains), the addition of our 665 novel genomes substantially increased genomic reads mapped for all 1,391 shotgun metagenomic samples from the HMP. By body site, the largest increases in reads mapped were observed for HMP samples from oral sites including the tongue dorsum and buccal mucosa (Fig. 4A and Table 1), which was expected since the “respiratory” sample category includes both oral samples and respiratory clinical samples collected through the oral route (see Tables S1 and S2). Besides gastrointestinal (GI) samples, these sites were two of the most numerous found in the existing database, indicating a notable absolute as well as relative increase in the amount of read mapping (Fig. 4B). While we did not specifically try to prioritize the fecal microbiome, the substantial increases in its characterization reflect both the strength of the prioritization process here for novel strains and the large diversity of the fecal microbiome within and between individuals, providing it more potential for increases in characterization of metagenomic shotgun reads. Although there were smaller increases for the anterior nares and posterior fornix, the skin samples collected as part of this study include many abscess, wound, and limb skin tissue samples, so these may not be well represented among the sample test sets used but will still be valuable for future studies.

**TABLE 1:** Average read mapping of samples grouped by body site to GenBank and novel HMSC genomes versus GenBank alone^*a*^

Body site	No. of samples	Avg no. of reads		
		Cleaned reads/sample	Mapping to GenBank	Mapping to GB + HMSC
Anterior nares	151	1,931,203	1,257,949	1,270,602
Buccal mucosa	241	10,521,737	8,276,060	8,588,770
Posterior fornix	137	3,876,399	3,212,985	3,218,331
Feces	319	88,431,094	60,971,330	61,628,060
Supragingival plaque	261	52,284,221	40,001,982	40,713,876
Tongue dorsum	282	78,327,283	57,097,893	59,722,717

a GenBank was considered the “baseline” database prior to this study’s addition of novel genome assemblies. GB + HMSC refers to GenBank and novel HMSC genomes.

Taxonomically, the largest increase in reads mapped was observed for bacteria from the phylum *Fusobacteria*. This phylum has historically been poorly characterized in databases, so our sequence contributions increased mapped reads by almost 30% (Fig. 4C). The phyla *Proteobacteria* and *Actinobacteria* showed modest increases in mapped reads, while the well-characterized *Firmicutes* and *Bacteroidetes* phyla showed only very small improvements. The improvements observed in read mapping for these taxa are likely related to the improvements that we observed in read mapping for the oral mucosa, which contains greater proportions of *Fusobacteria*, *Actinobacteria*, and *Proteobacteria* (26).

**FIG 5:**
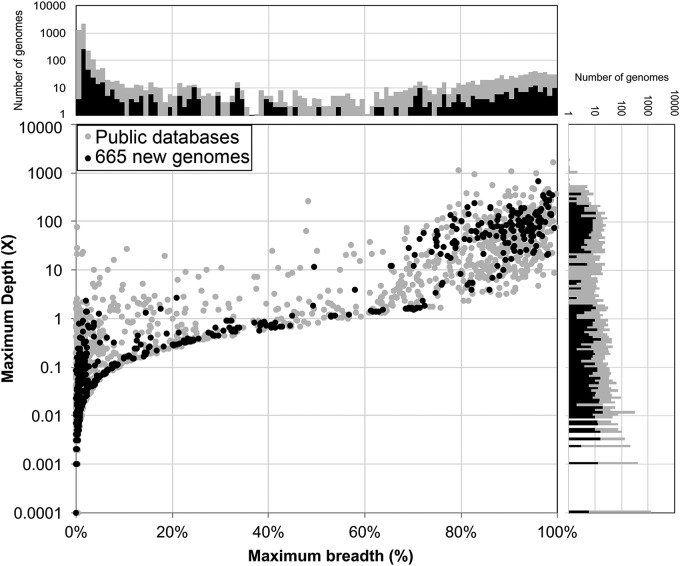
Novel genome assemblies (black) show increased representation in HMP samples, relative to sample size (>50% breadth, >1× depth, upper right quadrant of graph). Novel genomes are shown in black; public database genomes are shown in dark gray. Numbers are not normalized for database size.

This overall improvement is unsurprising given the high representation of our novel genomes among diverse HMP samples. Of the 665 (27.5%) novel genomes sequenced, 183 had high representation in at least one of the HMP samples (≥50% breadth and ≥1× depth). In comparison, only 12.5% of the public database genomes had high representation (*P* < 10^−5^ for enrichment of high-abundance strains) (Fig. 5).

There are several possible reasons for the enrichment of our novel genomes in the HMP data set relative to GenBank. First, our genomes were all sequenced from human samples, compared to many environmental samples that contribute to other public databases. For this reason, our results are particularly applicable to biomedical research endeavors. Second, our novelty by a phylogeny prioritization process, which selected a representative sequence from a cluster of similar sequences, emphasized variety in our rank list. Rather than simply sequencing all of the genomes that were determined to be the most novel, our process ensured sampling of novel genomes from many different taxa. Third, our selection of isolates from undercharacterized body sites broadens the types of samples that would find representation in a database. At the moment, HMP statistics show a database composition primarily of GI and urogenital cultures, with several other body sites having fewer than 10 reference genomes (27). For this reason, samples taken from body sites such as the eyes or respiratory system might contain genomes from our newly sequenced cohort but not from public databases.

If undertaken with focused sampling efforts, culturing and sequencing of isolates determined by our prioritization process could yield even greater improvement to known databases. Our isolates were taken from samples originally obtained from a variety of standard-of-care culture types from patients suspected to have an infection, thus resulting in a variety of sample qualities. Poor quality or technical workability sometimes eliminated what would have otherwise been a highly ranked isolate for sequencing. Additionally, our approach was still limited to those taxa that could be cultured in a laboratory setting using routine media commonly used in the clinical microbiology laboratory. There may be other taxa, especially among the variety of body sites in our study that are important but have heretofore not been sequenced due to their inability to grow in culture or inability to grow with the culture techniques used as part of routine clinical microbiology.

Our results demonstrate the benefit of a targeted approach to the sequencing and assembly of new microbial genomes. They also contributed 665 novel genomes originating from diverse human habitats to public databases that can be utilized by other studies. If more endeavors similar to this one are undertaken in the future, read mapping could be improved even more for microbial community samples spanning a variety of human habitats. As our understanding of the microbiome continues to improve and to enter the realm of therapy developments, such endeavors will increase in importance from both an academic and a clinical perspective.

## MATERIALS AND METHODS

### Sampling and culturing.

Clinical specimens were submitted to the Barnes-Jewish Hospital Clinical Microbiology Laboratory and were processed using culture media, incubation atmosphere, and incubation time as described in the clinical laboratory’s standard operating procedures for each specimen type. Bacterial isolates that morphologically resembled resident microbiota for the sample type cultured were selected, and a suspension of the microorganism was made for sequence-based analysis.

### Bacterial 16S rRNA genes were sequenced to identify novel bacterial strains associated with diverse habitats.

The full length of the 16S rRNA genes (16S) for each bacterial strain was obtained by sequencing three overlapping regions on the 3730 ABI platform. Primers used for the three amplicons were as follows: V1-V3, 27F (AGAGTTTGATCCTGGCTCAG) and 534R (ATTACCGCGGCTGCTGG); V3-V5, 357F (CCTACGGGAGGCAGCAG) and 926R (CCGTCAATTCMTTTRAGT); and V6-V9, U968f (AACGCGAAGAACCTTAC) and 1492r-MP (TACGGYTACCTTGTTAYGACTT). A total of 10,787 microbial isolates with 16S reads generated on the Sanger 3730 platform were analyzed. Following analytical processing and removal of chimeric 16S reads (28), a phylogenetic stepwise approach was undertaken to prioritize the potential novel genomes to be sequenced. Comparison to publicly available 16S rRNA gene sequences was performed using a BLAST database that contained nonredundant 16S rRNA gene sequences from SILVA v.115 (29) and Ribosomal Database Project (RDP) (30) training set 9. The 16S rRNA genes from the Washington University Strain Collection (WUSC) were subjected to a BLAST search against this database, and any sequence with ≤97% identity and/or ≤90% coverage against any of the strains in the existing databases was considered potentially novel (225 total “novel” strains). Following this initial processing, of the 10,787 starting strains, 4,546 sequences (from 3,798 unique samples, due to the sequencing of technical replicates) met three criteria to advance to further analysis: successful 16S assembly (using the One Button Velvet assembly pipeline, version 1.1.06 [31]), metadata completeness, and successful SILVA/RDP classification.

To further prioritize isolates, we performed phylogenetic analysis to avoid repeatedly sampling very similar isolates and to preferentially select novel sequences, such as those similar to known sequences but isolated from a different body site. The 16S rRNA genes from the complete bacterial genomes from the HMP (whole-genome shotgun-based sequencing; inclusive of the 4 HMP sequencing centers; McDonnell Genome Institute at Washington University School of Medicine, J. Craig Venter Institute, Baylor College of Medicine, and the Broad Institute) and the Greengenes GOLD database were clustered into a nonredundant database by clustering all sequences using 99% identity and 95% coverage (32), with the longest sequence per cluster used as a representative. The WUSC 16S rRNA genes from the 3,798 unique samples were then clustered with these representative sequences at 99% identity over 95% length. A total of 247 WUSC samples were determined to be contaminated due to the presence of multiple sequence replicates from the same sample being present in different clusters, leaving 3,551 samples for downstream prioritization.

The taxonomic classifications were used to split the sequences into taxonomic groups manageable for manual evaluation, by constructing phylogenetic trees for each taxonomic group using mothur (33). The 15 resulting phylogenetic trees were visualized using iTOL (34), and 16S rRNA sequences were considered to be novel by phylogeny if they did not cluster with the 16S rRNA gene of a sequenced bacterial genome or if they originated from different body sites than the sequenced isolate (262 samples). For simplicity in the prioritization, body site information was collapsed into a broader category when it was more detailed (e.g., abscesses from any body site were collapsed to “Abscess”).

The top available samples were prioritized for sequencing according to (i) all novel samples first (20 final samples sequenced); (ii) the top-ranked sample within each novel by phylogeny cluster (88 samples); (iii) known cluster top representatives (51 samples); (iv) novel by phylogeny samples from unique isolation sources but which were not the top ranked in their cluster (only up to 5 per cluster, since there are some very large clusters; 128 samples), and (v) novel by phylogeny samples, sorted by within-cluster rank, selecting for samples from rare or desirable body sites (355 samples); and (vi) known, unique isolation sources (23 samples).

Overall, from the 10,787 starting 16S sequences in the WUSC database, 4,546 with SILVA/RDP classifications and available metadata were selected (representing 3,551 unique samples; see Table S1 in the supplemental material), and 665 samples were prioritized for genome sequencing based on availability, strain novelty compared to existing databases, and body site of origin, with a strategic approach used to avoid sequencing similar samples repeatedly (Table S2).

### Genome sequencing, assembly, and annotation.

DNA extraction and whole-genome shotgun sequencing on the Illumina GAIIx platform was performed as previously described (26). One Button Velvet (version 1.1.06) (31) was used to assemble the genomes, and HMP cutoffs and settings (7) were applied for a sequence to have enough contiguity to be advanced to the annotation level. We screened for contamination by subjecting the assembled supercontigs to a BLAST search against a database of all HMP strains sequenced in-house using BLASTN. If supercontigs from the same strain had a top hit against 2 different genera, this strain was considered contaminated and discarded. Those that failed were discarded from the pipeline. The GC% plot was reviewed as an additional measure to detect mixed data from different sources. Any contigs identified as contaminated were removed, and the resulting assemblies continued into the annotation pipeline. Annotation was performed as previously described for the HMP reference genomes (26), and taxonomic validation was performed by checking the 16S gene against the reference database and ensuring that the taxonomic identity of the majority of the predicted genes (by BLASTp against GenBank’s bacterial NR database [35]) matched the identity determined by the SILVA/RDP matching methods. Genome completeness was tested using BUSCO, version 4 (16).

### Analysis of HMP metagenomics shotgun data.

To quantify the value of our 665 newly assembled Human Microbiome Sequence Collection (HMSC) genomes, we mapped 1,391 HMP samples from 6 body sites against a baseline bacterial database containing all GenBank complete and drafted bacterial genomes (circa March 2016) (26). We then mapped the same samples to the GenBank database with our 665 HMSC genomes added.

When mapping sample reads to GenBank database entries, we used only GenBank entry genomes with annotation. In cases in which there were multiple entries for a single taxon, we used only the longest representative. The HMP samples used as queries were chosen from 6 body sites: anterior nares, buccal mucosa, posterior fornix, stool, tongue dorsum, and supragingival plaque (26). Outliers with regard to sample read counts (“cleaned” read counts per sample) were filtered by applying an upper limit of 3× standard deviation of read counts above the mean per sample for each body site. A lower cutoff was also set to remove roughly the bottom 5% of the smallest samples. This resulted in 1,391 samples being used as queries.

Contaminating human reads in query samples were masked using BMTagger (ftp://ftp.ncbi.nlm.nih.gov/pub/agarwala/bmtagger). Duplicate reads were marked and removed using a modified version of EstimateLibraryComplexity, a tool from the Picard package (http://picard.sourceforge.net/index.shtml). Low-quality sequences were then trimmed away using a modified version of the script trimBWAstyle.pl (J. Fass, The Bioinformatics Core at UC Davis Genome Center). This script removed bases with a quality of 2 or less from the ends of reads, an indicator of uncertain quality as defined by Illumina’s End Anchored Max Scoring Segments (EAMMS) filter. After masking and quality trimming, reads with fewer than 60 consecutive non-N bases were removed. The cleaned reads for each sample that passed our filters were then mapped against both databases (GenBank [GB] bacterial genomes and GB bacterial genomes plus HMSC genomes) using Bowtie 2 v2.2.5 (default settings: end-to-end mode, zero mismatches allowed in seed alignment during multiseed alignments, multiseed length 22).

### Data availability.

All assembled and annotated genomic sequences and metadata are deposited in NCBI’s GenBank (25). All BioProject, BioSample, and accession identifiers (IDs), metadata, and assembly statistics are provided per sample in Table S2 in the supplemental material.
